# Recent Advances in Biology, Host and Microbe Interactions of the Human Sexually Transmitted Parasite *Trichomonas vaginalis*

**DOI:** 10.3390/ijms262412015

**Published:** 2025-12-13

**Authors:** Desmond L. Seybold, Gregory P. Contreras, Jia-Feng Chang, Ting-Yu Yeh

**Affiliations:** 1 Department of Biological Sciences, University of Maryland Baltimore County, Baltimore, MD 21202, USA; desmonds2023@gmail.com; 2 Agricultural Biotechnology Laboratory, Auxergen Inc., Riti Rossi Colwell Center, Baltimore, MD 21202, USA; greg@auxergen.com; 3 Institute of Marine and Environmental Technology, University of Maryland Baltimore County, Baltimore, MD 21202, USA; 4 Auxergen Srl, Technopolis Science and Technology Park of the University of Bari, 70010 Valenzano, Italy; 5 Division of Nephrology, Department of Internal Medicine, Taoyuan Branch of Taipei Veterans General Hospital, Taoyuan 330, Taiwan; 6 Department of Nursing, Yuanpei University of Medical Technology, Hsinchu 300, Taiwan

**Keywords:** *Trichomonas vaginalis*, cytoadherence, host–parasite interaction, immune response

## Abstract

Trichomoniasis is the most common non-viral sexually transmitted infection in humans, with over 200 million people affected each year. This disease is associated with pre-term birth, low birth weight, and premature membrane rupture. Its causal pathogen, *Trichomonas vaginalis* (TV), is a prevalent sexually transmitted protozoan parasite that infects the urogenital tract through cytoadherence. TV infection alters TV gene expression and induces host immune responses, while TV-secreted exosomes carry RNA and protein cargoes that mediate extracellular signaling. This review summarizes recent discoveries of molecules that interact with host receptors involved in cytoadherence. We also discuss human innate and adaptive immune responses to TV infection via a variety of inflammatory mediators. Recent research on concurrent or endosymbiont relationships of TV with other urogenital microbes and cancers, is also examined. These studies not only highlight the necessity of understanding host–microbe interactions in TV pathogenesis but also provide a crucial insight into potential therapeutic targets of nitroimidazole-resistant TV strains.

## 1. Introduction

Trichomoniasis is the third most common sexually transmitted infection in humans [1]. This disease is caused by the most common non-viral sexually transmitted organism in humans, *Trichomonas vaginalis* (TV) [2,3]. TV affects over 200 million people a year, with 150 million people between 15 and 49 years old newly infected in 2020 [2,4]. Many undiagnosed cases likely persist, contributing to ongoing, unrecognized transmission. Symptoms of TV infection may include vaginitis and cervicitis, with variation from case-to-case [5,6]. Vaginal discharge, urethral discharge, and soreness are commonly experienced when symptoms are present [2]. Greater numbers of women with TV (~50%) are symptomatic, than infected men (~25%) [7]. People with trichomoniasis typically exhibit mild symptoms, which have historically led to underdiagnosis of the condition and little research being performed on the topic [5].

The parasite is an anaerobic, flagellated protozoan that adheres to the epithelial cells lining the human urogenital tract [4,8]. Humans are the only known hosts for the parasite [3]. TV exists only in a trophozoite phase in situ, with no cystic phase, though it may enter a pseudocystic stage when taken outside the body [5,9]. TV occupies the female cervix and vaginal tract, as well as the male upper and lower urogenital tract. TV can be transmitted through semen, urethral discharge, urine, and prostatic fluid [10].

The immune response to TV involves many proinflammatory cytokines from leukocytes, neutrophils, macrophages, and other host immune cells. TV possesses many immune evasion systems, allowing it to evade antibodies and elicit host responses that permit the parasite to advance further into the body. Immune responses include both the innate and adaptive systems, involving innate systems in particular at mucosal surfaces.

TV interacts with a large community in the vaginal microbiome, including bacteria, fungal cells, and yeast [11]. TV works in tandem with endosymbiont dsRNA viruses of genus *Trichomonasvirus* (TVV) [4]. *Lactobacillus* helps the host mitigate TV infection, while two *Mycoplasma* species, *Mycoplasma hominis* and *Candidatus Mycoplasma girerdii*, contribute to the parasite’s pathogenesis [12]. *Gardnerella vaginalis* is the primary causal agent of bacterial vaginosis (BV) and is associated with greater susceptibility to TV in patients [13]. Alongside BV, trichomoniasis can increase the host’s susceptibility to HIV, prostate and cervical cancer [14,15].

In recent years, multiple new reviews focusing on host–parasite interactions of TV have been published [16]. Popular treatments related to trichomoniasis, namely metronidazole treatment, were found to be less effective than previously assumed, and research into alternative treatments is limited. The availability of new technology to investigate these issues to date is limited despite the pervasiveness of the disease. In this review, we focus on the mechanistic effects of TV adherence on the host cell and the influence of TV infection on human immune and inflammatory responses. We also discuss the relationships between TV and other microbes and diseases inhabiting the vaginal microbiome. The information reviewed here may contribute to potential treatments of drug-resistant TV strains.

## 2. Part 1: Cytoadherence

TV cytoadherence is executed in multiple stages. The trophozoite first transforms into an amoeboid form upon contact with the host [17]. This amoeboid form allows the parasite to cover the targeted epithelial cell through the subsequent extension of filopodia and pseudopodia over its surface [18]. TV then synthesizes and expresses adhesins and uses its axostyle and surface lipoglycans to secure and mediate the infection [17,18]. Cytoadherence utilizes primarily contact-dependent cytolysis via adhesins [16]. Finally, the aggregation of many parasites on the epithelial wall forms clusters over the surface of the urogenital tract [19]. In the absence of host cells in vitro, clusters become fragile, and TV may form a monolayer instead [20]. In such conditions, TV may not utilize all the cellular restructuring processes previously stated, nor the typical extracellular vesicles to interact with other TV cells [20].

### 2.1. TV Genetic Regulation, Epigenetic Regulation, and Post-Translational Modifications

The TV genome undergoes transcriptional changes when infecting the host, although currently, few core regulatory elements and transcription factors have been identified in this process [21]. TV transcription factor IBP39 binds to the Initiator (Inr) element, the most important core promoter element of TV, allowing IBP39 to interact with both DNA and RNA polymerase II to control basal transcription in TV [21]. The highly conserved TATA box sequence is only found in some promoters in the TV genome; however, Parra-Marin et al. identified TVTBP1 and TVTBP2, putative TATA box binding proteins that are expressed under normal growing conditions, and which interact non-specifically with DNA [21].

The epigenetic differences between TV strains can greatly influence their adhesive ability. Histone modification, such as H3KAc, mediates both transcription and pathogenesis. H3KAc is enriched in nucleosomes around the transcription start site of genes BAP1 and BAP2 in adherent strains, while low-adherence strains have less acetylation [22]. The binding of the Inr-binding protein also depends on the initiator’s histone acetylation state. Treatment with Trichostatin A, a histone deacetylase inhibitor, was also found to increase parasite aggregation and adherence to the host [22]. Another study found that H3K9, H4K5, H4K8, and H4K16 were acetylated more in the less adherent T1 strain than the more adherent TH17 subtype of CD4+ T cell trophozoites, and H4K12 was more acetylated in TH17 than in T1 [17].

Little is known about the distribution of phosphorylated proteins over the life cycle of TV. A study in 2013 profiled the proteins in the trophozoite, amoeboid, and pseudocyst stages of TV, finding 21 proteins in all stages, 29 in two different stages, and 32 stage-specific proteins, out of 82 unique proteins from 93 phosphopeptides [23]. Phosphorylation of these proteins may play a role in the transformation of TV between these stages. Very little research into TV phosphoproteomics has been conducted thus far [23].

At least 363 S-acylated proteins regulate processes such as protein transport, metabolism, pathogenesis, and signaling. Palmitoylation could modify proteins that regulate pathogenesis through parasite aggregation and adherence [24]. In addition, 7 novel DHHC domain containing Palmitoyl Acyl-Transferase-like proteins may act as active enzymes, and many surface and secreted virulence factors are palmitoylated [24].

### 2.2. Exosomes and Extracellular Signaling

**Figure 1:**
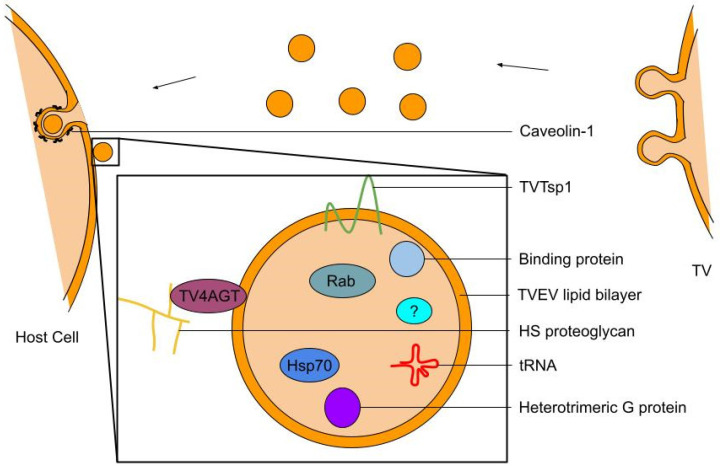
Cargo of *T. vaginalis* Extracellular Vesicles (TVEVs). TVEVs (enlarged view, below) carry the depicted cargo, among other secretion including potentially unknown cargo. TV secretes EVs through caveolin dependent endocytosis, which increases pro-inflammatory cytokine levels, enhances TV adherence, and degrades epithelial junctions [31].

Extracellular signaling in cytoadherence and the infection process use exosomes to transport factors. Exosomes are 30–100 nm extracellular vesicles (EVs) secreted by multiple cells in the vaginal microbiome, from a variety of players, and facilitate a significant portion of cell signaling with their cargo (Figure 1) [25]. TV-secreted EVs (TVEVs) can induce changes in the host cell and promote the parasite’s colonization of the host cells [25]. TVEVs are used for parasite-parasite interactions, as well as interspecies interactions within the vaginal microbiome. TVEVs are physically similar to mammalian exosomes, and possess similar protein components [25]. TV also produces microvesicles, separate membrane-shed vesicles likely involved in intercellular communication [26].

TVEVs originating from well-adhering B7RC2 strain, can strengthen the adherence of poorly adhering parasites, such as the laboratory G3 strain, while weaker parasites cannot strengthen the stronger cells [27,28]. RNA and surface receptors contained in TVEVs can signal antigens, with which RNA could also be used as a biomarker for infection [25]. TVEVs may not only carry genetic information such as through RNA but could also contribute to protein–protein interactions [27]. TVEVs contain core, conserved exosomal proteins as well as parasite-specific proteins [25]. One meta-analysis found 16 core proteins, including tetraspanin, catalytic activity and binding, Rab, and heterotrimeric G proteins contained in the TVEVs [29] (Figure 1). With this capability in mind, the primary function of TVEVs may be to provide greater surface area for long-distance contact-dependent protein, lipid, or polysaccharide interactions. The vesicle membrane enables the lateral diffusion and sorting of membrane ligands on the cell surface, promoting avidity-driven effects and co-stimulatory signals for the activation of immune responses. This includes mechanisms such as vesicle-induced receptor sequestration (VIRS), which concentrates TVEV-presented ligand receptors on host cells into signalosomes, increasing their signaling capacity [27].

TVEV production is modulated by pathways such as the Endosomal Sorting Complex Required for Transport (ESCRT). Modifying the ESCRT complex, such as by overexpressing VPS32 can increase adherence to host cells [27]. In addition, TVEVs can be generated in an ESCRT-independent manner, such as through tetraspanin-mediated pathways [30]. The effects of TVEVs and their roles in cell–cell communication depend on the uptake and release of vesicle cargoes. TVEV uptake is dependent on cholesterol and caveolin-1. Subsequent internalization by the recipient cell uses clathrin-independent, lipid raft-mediated endocytosis. To release cargoes inside the cell, TVEVs interact with host glycosaminoglycans (GAGs) on proteoglycans of the recipient cell membrane [31]. TVEVs bind to heparan sulfate (HS) GAGs in particular [32]. 4-α-glucanotransferase (TV4AGT) ligands on the TVEV surface bind to HS [31]. TVEVs can induce the formation of filopodia and cytonemes, long, thin, actin-rich protrusions, through paracrine signaling between strains. TVEVs isolated from more adherent strains of the parasite can prompt the formation of these membrane protrusions on other parasites during the aggregation and adherence process [33]. TVEVs upregulate the expression of parasite membrane proteins in each other [28]. Furthermore, TVEVs upregulate and directly transfer adherence factors promoting host cell colonization, such as the recently characterized heteropolysaccharide binding protein 2 (HPB2) [28]. In addition, TVs hold cadherin-like proteins such as TVAGG3_0583720, a suspected mediator of host cell adherence that is transferred between cells in the secretion and uptake of EVs [28].

### 2.3. Factors Involved in TV Cytoadherence

#### 2.3.1. Adhesins

**Figure 2:**
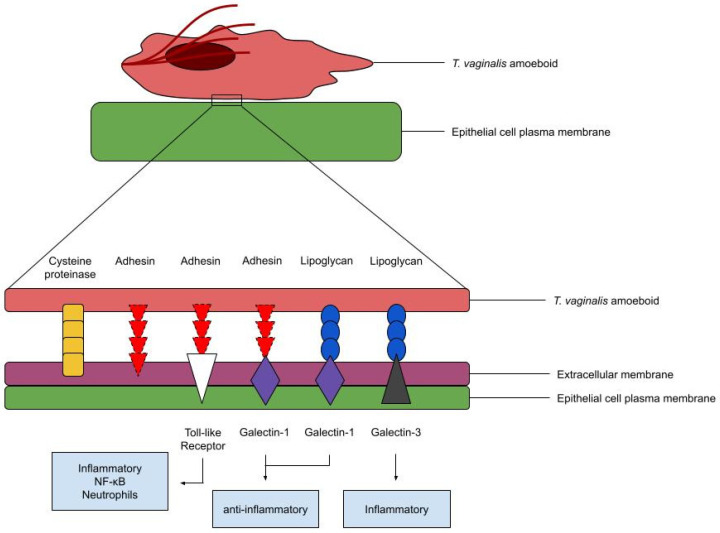
Adherence Factors of *T. vaginalis*. The proper designation for adhesins as adhesion proteins, malic enzymes, or otherwise is debated [37]. Cysteine proteinases may be secreted or present on the surface of the parasite. TLRs activate pro-inflammatory cytokines, NF-κB pathways, and lead to neutrophil recruitment [1]. Galectin-1 suppresses inflammation, while galectin-3 activates pro-inflammatory signals [48].

Cytoadherence acts through many TV and host factors. The parasite employs adhesins to cover and attach to the plasma membrane of its host cell. These adhesins interact with receptors such as galectin-1 or Toll-like receptors (TLRs) (Figure 2) [17]. Galectins represent an evolutionarily conserved family of glycoproteins with one or two carbohydrate-recognition domains that bind to β-galactosides [34]. Infection likely consists of a galectin-independent phase, and galectin-dependent swarming phase in TV cytoadherence [17]. In Th17 cells, TV infection induces secretion of galectin-1 and -3 from epithelial cells which bind to the parasite, followed by engulfment of galectin-3 in the adherent parasite’s lysosome [17]. Alternatively, adhesins may adhere to the surface of another inhabitant of the vaginal environment instead of the host. Aside from its role in aggregation, the significance of galectins in TV infection remains to be studied [17].

Adhesins appear to lack strict specificity and are capable of binding to a variety of cell types as well as non-cellular surfaces, notably medical equipment [35,36]. There is controversy over whether adhesins bind specifically at all. Others raise questions over the true designation of adhesins as adhesion proteins, with some suggesting they are better classified as malic enzymes [37]. Adhesins do not attach to laminin and fibronectin of the extracellular matrix [38]. TV uses five primary adhesins: AP120, AP65, AP51, AP33, and AP23 to mediate adherence to the vaginal epithelium [39]. Most of these adhesins are upregulated by iron, a virulence factor and essential mediator of TV growth [5]. Adhesins are synthesized and transported upon TV transformation from trophozoite to amoeboid [40]. The adhesins are also characterized as hydrogenosomal enzymes PFO (AP120), ME (AP65), α-SCS (AP33), and β-SCS (AP51). These adhesins may have additional moonlighting functions, though α- and β-SCS subunits likely do not, as they appear to target only hydrogenosomes [41]. For example, PFO A shares sequence homology with AP120 and is encoded by the same gene. PFO is a surface-associated cell-binding protein lacking enzyme activity, involved in adherence to host cells, in iron-rich conditions [42].

The protein AP65 is one of the most important of the five adhesins. Anti-AP65 serum IgG antibodies inhibit TV cytoadherence, unlike those for the other adhesins [43]. AP65 lacks a covalent anchor motif and is released extracellularly, where it binds to both TV and host cells. AP65 can be taken up by host cells to upregulate immune genes [39]. Earlier studies found that laboratory-adapted TV synthesized lower amounts of adhesins, which was correlated with a loss of the ability to upregulate synthesis with iron [44]. AP65 and AP51 are likely heme- and hemoglobin-binding proteins in addition to adhesion proteins [45]. TV AP65 modulates parasite pathogenicity by allowing for cell adherence, inhibition of host cell proliferation, and induction of host cell apoptosis. Zhang et al. reported that TVAP65 binds to VK2/E6E7 epithelial cells, and interacts with 13 protein molecules [46]. One of the proteins, Bcl-2 interacting protein 3 (BNIP3), plays a role in TV pathogenesis [46,47]. The pathogenicity of TV decreases with anti-rTVAP65 PcAb passive immunization or blocking the TVAP65 protein. AP33 also interacts with BNIP3 to help mediate host–parasite adhesion and pathogenicity [46]. AP33 could be used as a vaccine candidate antigen to induce cell-mediated and humoral immunity, as it triggers a high antibody response and has a high antigen index [47].

#### 2.3.2. Lipophosphoglycan (LPG)

Few surface molecules are confirmed to be involved in parasite attachment to epithelial cells, with the LPG adhesion molecule being the most studied [49,50]. LPG is composed mostly of carbohydrate and lipid without any peptide [51]. Part of the LPG, the ceramide phospho-inositol glycan core, ligates to galectins [51]. TV LPG contains poly-N-acetyllactosamine repeats [52]. Unlike other glycosylphosphatidylinositol-anchored molecules, no mannose is found in TV LPG, unlike LPG found in other parasites. TV LPG is not structurally modified during its development [51,53]. LPG immune responses vary across species [54]. LPG exists on the surface of TV in the glycocalyx, and binds to galectin-1 and -3 on the host cell [48]. Galectin-1 is the primary receptor of TV LPG on epithelial cells [55,56]. When LPG binds with galectin, it prompts cytokine release. In the presence of LPG, host cells upregulate pro-inflammatory mediators, while TV can also trigger an immunosuppressive response [57]. TV LPG is the most abundant glycoconjugate on the membrane surface, and anchors the parasite to epithelial cells via inositol-phosphoceramide (CPI-GC) [57].

#### 2.3.3. Cysteine Proteinases (CPs)

CPs are virulence factors expressed in TV to perform a wide range of functions and are necessary for cytoadherence [58]. One example is the recently studied TV LEGU-1, Legumain-Like CP, which is a high-affinity enzyme important to host attachment. Cytoadherence was reduced by 80% when TV LEGU-1 was inhibited by an aza-peptidyl Michael acceptor, a new class of irreversible inhibitors that are highly potent and specific for clan CD CPs [59]. CP secretion via a lysosome-dependent pathway may contribute to cytopathic effects [60]. Studying the CPs could yield new therapeutic targets [59]. A new ELISA assay for detecting TVCP39 antibodies with high specificity and sensitivity was recently developed [61]. Knocking down TVCP39 reduced the parasite’s viability, adhesion, and its ability to inhibit cell proliferation and induce apoptosis. It also reduced TV promotion of human papillomavirus (HPV) infection. Anti-rTVCP39 polyclonal antibody blockade or passive immunization effectively reduced *T. vaginalis* infection and vagina damage [62]. One CP endogenous inhibitor, Trichocystatin-2, may represent a potential therapeutic target [63]. TV binds to the mucin that shields epithelial cells and degrades them with CPs called mucinases to allow the parasite access to the epithelium [64]. Proteolytic activity can also degrade immunoglobulin to participate in immune evasion, as well as disrupt cytokine and receptor activity [65,66]. Some CPs degrade ECM components to facilitate the attachment of the parasite, such as CP65, which degrades type IV collagen and fibronectin [67]. CPs also participate in hemolysis, which may allow TV to replenish its iron [64,68]. CP activity depends on the pH and temperature in the urogenital tract, like many other compounds involved in trichomoniasis [69]. Other microbial factors may affect TV cytoadherence activity indirectly via CPs. For example, CPs are neutralized by H2O2 produced by Lactobacilli [70]. TV virus (TVV) infection increases expression of CPs and P270, which allow TV to degrade hemoglobin, fibronectin, collagen IV, and the basement membrane of host epithelial cells [71].

#### 2.3.4. Cytoskeleton

Surface adhesion molecules act in conjunction with parasite cytoskeleton behaviors to initiate cytoadherence. Cytoskeleton reorganization is important for flagellate-amoeboid transformation and consequential cytoadherence [17]. Microtubules, microfilaments, and actinin dictate the structure of the parasite as it attaches to its host. α-actinin is a key component of cytoskeletal restructuring in TV cytoadherence and facilitation of cell movement, as well as being a common immunogenic protein. This property makes α-actinin a strong candidate for TV vaccines [72,73].

The newly discovered virulence factor TVTIM is a surface-associated protein under high glucose conditions and mediates specific binding to laminin and fibronectin. The enzyme is encoded by two genes: TVtim1, which is overexpressed with restricted glucose, and TVtim2, which is overexpressed with abundant glucose [74]. GAPDH, typically a glycolytic enzyme, moonlights as an adhesion protein by binding to fibronectin, plasminogen, and collagen, with its expression being upregulated by iron [75].

#### 2.3.5. Iron

The influence of iron is key to TV adhesion, which is largely mediated by iron-dependent surface adhesins [76]. All five primary surface adhesins except AP51 are upregulated by the presence of iron [77]. Iron regulates metabolism, host attachment, and proteolytic degradation, also facilitating immune evasion and cytopathology [78]. The iron pathway allows for actin remodeling in phagocytosis, exacerbating cytopathic effects [78]. Iron transiently triggers PIP2 signaling in the restructuring of actin in TV during infection, regulating contact-dependent and independent cytotoxic activity [78,79]. Iron also regulates TV PI4P5K translation, lysosomal degradation, and TV PI4P5K membrane trafficking using TV Arf220 ribosylation factors to control PIP2 production [78]. Arf220 also increases TV intracellular calcium levels by increasing PIP2 production [78]. In TVV-infected parasites, the localization of the major immunogen P270 is modulated by iron levels [80]. Other metals, such as zinc, can influence proteins involved in cytoadherence in a similar way to iron [64]. CPs can elicit iron responses, while iron levels can also regulate CPs. Iron can up or down regulate CPs depending on its identity, such as CP4 or CP65, respectively. Arroyo et al.’s study provides a list of CP iron responses. Post translational modifications to CPs are also regulated by iron [69].

#### 2.3.6. Other Proteins

Many additional adherence factors play roles in cytoadherence, including BspA proteins and rhomboid serine proteases. BspA proteins are surface adhesion proteins with 23 amino acid long leucine-rich repeats [81]. The vast BspA-like gene family highlights functional diversity supported by its structural organization and transcriptomic data [19]. BspA and Pmp variants are suspected to be associated with increasing host cell adhesion, though how remains unclear. Pmps might recognize specific host cell structures, leading to the endocytosis of host material. These gene families are present in all trichomonads, but they are massively expanded in TV. How the families were acquired is debated. Studies towards determining their origin indicate they may have been acquired through ancient acquisition from mucosal-dwelling prokaryotic microbiota, or independent acquisitions through HGT [81]. Two TV rhomboid proteases are catalytically active: TVROM1 and TVROM3. TVROM1 has a role in promoting TV attachment to host cells and lysis of host cells [82]. Other relevant factors include metalloproteases and GP63 proteases. The hypothetical TVAG_157210 (TVAD1) protein may also be used in adherence [32]. Another cadherin-like protein designated TVAG_393390 mediates parasite–parasite and host–parasite adherence, the overexpression of which leads to increased aggregation, which is enhanced by calcium [43]. The serine/threonine protein phosphatase, PP1γ, is involved in proliferation and attachment to the host. PP1γ-dependent dephosphorylation of the cytoskeleton is involved in the early stages of the host–parasite interaction and may represent a potential drug target [76].

## 3. Part 2. Human Immune and Inflammatory Responses to TV Infection

In response to TV infection, the human body activates multiple immune and signaling pathways to defend itself. TV infection upregulates many proinflammatory mediators, including IgA, IgG, and Th1 cytokines [83]. The immune response to TV uses a variety of cytokines (IL-8, IL-6, IL-1β, TNF-α, MIP-3α/CCL20, MCP-1/CCL2) in host–cell interactions.

### 3.1. NLRP3 Inflammasome

TV induces NLRP3 inflammasome activation in macrophages, leading to IL-1β production, along with the secretion of other cytokines such as IL-8, which recruits neutrophils [1,84]. NLRP3 activation involves host cell detection of extracellular ATP via P2X7 receptors and potassium efflux, which leads to caspase-1 activation and the processing of pro-IL-1β into IL-1β. The inflammasome induces macrophage pyroptosis via caspase-1 cleavage of the gasdermin D protein, which forms pores in the host cell membrane [84].

### 3.2. Neutrophil Trogocytosis

In addition to epithelial cells, immune cells such as dendritic cells, macrophages, and neutrophils also encounter parasites. Inflammatory mediators are secreted in response to epithelial cells, macrophages, and neutrophils that first arrive at the infected site [1,3]. The immune system mainly employs neutrophils to defend the body from TV [85]. TV adherence to epithelial cells prompts the release of IL-8, and neutrophil recruitment by emerging from the bloodstream and migrating into the tissue [52]. The inflammatory mediators activate the epithelium to trigger recruitment of additional neutrophils from extravasation to diapedesis [1]. Once locating the infected cells, the neutrophils enclose and trogocytose the parasite, physically taking small “bites” out of the membrane until it is rendered inert [85]. Leukotriene chemical mediators such as LTB4 can also support the transportation of neutrophils and continued attack of the parasites [3]. Trogocytosis is the method of consumption or lysis used by neutrophils to degrade TV, as opposed to phagocytosis or neutrophil extracellular traps (NETosis). Trogocytosis is dependent on neutrophil serine proteases and human serum factors and is mediated by antibody–fragment crystallizable (Fc) receptor interactions [86].

### 3.3. Reactive Oxidative Species (ROS) and NO

TV stimulates the production of IL-8, which is mediated through the NF-κB and MAP kinase signaling pathways, along with leukotrienes, reactive nitrogen intermediates, and inducible nitric oxide synthase (iNOS) in leukocytes [52,87]. Macrophages and monocytes produce ROS and NO, while secreting various cytokines to activate additional immune responses. Activated phagosomes release ROS, which is also involved in NETosis. TV can stimulate immune chemoattractants such as superoxide anions and NO, from various host immune cells, particularly macrophages [88]. ROS production in macrophages, monocytes, and neutrophils through pathways such as the NF-κB pathway leads to the production of iNOS and the release of cytokines [89]. In attacking the parasite, it is also possible for the neutrophil to damage the epithelium [1].

### 3.4. Extravasation

Macrophages produce the cytokine tumor necrosis factor (TNF-α), which recruits immune cells, and is upregulated in response to TV infection as a proinflammatory signal [90]. Extravasation is mediated by selectins on the epithelium, upregulated by IL-1β and TNF [1]. These factors slow the motion of passing neutrophils in the capillaries to a roll, as they grasp the epithelial cells with low-affinity selectins of their own [1]. Nearby inflammatory cytokines act alongside the selectins to instruct the neutrophils to activate high-affinity intercellular adhesion molecules (ICAMs), fully halting their passage and allowing for diapedesis into the tissue [1].

### 3.5. Interferon-Induced Tetrapeptide Repeat (IFIT) Family Proteins

TV infection also induces autophagic flux and IFIT overexpression to modulate inflammatory responses. IFIT3 positively regulates downstream IL-8 and IP-10/CXCL10 secretion. IFIT expression is suspected to be downregulated with autophagy. Blocking autophagosome formation via si-LC3B increases IL-6 and IP-10 levels while reducing IL-8 secretion. si-LC3B enhanced IFIT expression, suggesting that autophagy negatively regulates IFIT expression. It is suggested that autophagosome accumulation may counteract inflammatory responses to TV through unknown mechanisms. It is also possible that residual autophagosomes may continue to modulate inflammatory responses, preventing sharp increases in IL-6, IL-8, and IP-10 levels. Very little research has been performed on this subject so far [91].

### 3.6. TLR3 Signaling

TLR3 pathways are activated in another inflammatory cascade [92]. TVEVs induce secretion of CXCL1, IL-6, IL-8, and MIP-1β, among other inflammatory cytokines in macrophages and ectocervical cells [92]. TVEVs induce TLR3 overexpression to activate the NF-κB/NLRP3 pathway in macrophages and upregulate TLR3-regulated PI3K and NF-κB pathways, while simultaneously suppressing p38 MAPK and ERK pathways. TVEVs also upregulate MICB and TRAF3IP2 in the inflammatory cascade, which are both positively regulated by TLR3 [92]. The antibiotic SALF suppresses TV-induced secretion of proinflammatory cytokines by acting through the p38 and NF-κB pathways to inhibit gene expression [93].

### 3.7. Adenosine Signaling

Adenosine signaling by TV-stimulated neutrophils possesses the most abundant purinergic receptor and can lead to an increase in ROS and IL-8 levels [88]. ROS and ER stress responses to TV infection can induce apoptosis in cervical cancer cells through the ER-mitochondrial crosstalk IRE1/ASK1/JNK/Bcl-2 family protein pathways [94]. Adenosine protects host cells from parasite-induced cytotoxicity. TV relies on its hosts to salvage nutrients and adapts to their absence in part by altering adenosine metabolism [95]. Deprivation of adenosine results in enhanced extracellular nucleotide hydrolysis of host cells, using NTPDases. In these conditions, there is an increase in ATP, ADP, and AMP hydrolysis. This mechanism may help alleviate cytotoxic concentrations of extracellular ATP in TV [95]. The reliance of TV on host nucleosides makes nucleoside metabolism a drug target. Various nucleobase transporters have been found. The main broad specificity carrier seems to be TVagENT3, which displays high affinity for uridine and cytidine and purines [96].

### 3.8. Adaptive Immunity

The most effective host response to TV infection is primarily carried out through innate immunity systems, although adaptive immunity contributes to cellular defense and presents potential targets for therapeutic intervention. TV can lyse B cells and T cells, with a preference for B cells [97]. Antibodies use neutralization, opsonization, and complement activation to tag pathogens for destruction. IgA and IgG are the most relevant immunoglobulins to trichomoniasis, and are upregulated in response to the disease, while the levels of the others do not change significantly [6,98]. To evade antibodies, TV can degrade immunoglobulins using CPs, antigenic variation, and killing antibody-producing B cells [72]. Specific antibodies target LPG, α-actinin, migration inhibitory factor (MIF), pyruvate: ferredoxin oxidoreductase (PFO), legumain-1 (TVLEGU-1), adhesins, and CPs [72].

## 4. Part 3. Interactions with Other Diseases

The evidence that TV infection is associated with other high morbidity diseases has increased efforts to diagnose and treat TV [5]. The clear influence of diseases on the symptoms of trichomoniasis necessitates the investigation of interactions between TV cohabitants. Alongside the parasite, the environment is home to bacteria beneficial to the host, and yeasts, among others [99,100]. TV infection has an impact on a wide range of other diseases, from HIV and cancer to bacterial vaginosis (BV) [31,101]. Interplay between the diseases, which have competing effects on each other, leads to much complexity in the manifestation of TV. For example, HIV-1, BV, and TV all have a positive association together, while yeast levels decrease with Trichomoniasis [100]. Many of these interactions make appearances with significant correlation, providing valuable notice for diagnosis and insight as to the pathology of harmful diseases. TV is associated with the community state type IV (CST-IV) microbiome, defined by the absence of *Lactobacilli* and a dense population of anaerobic bacteria [102]. TV may or may not be the causative factor for the development of these microbiome populations [102]. This biome is conducive to pathological conditions such as BV, HIV, and preterm birth, which may hold responsibility or partial responsibility for the CST-IV designation [102]. These organisms alter the expression of tight junction proteins, particularly occludin, IL-6, and TNF-α [102]. Paracellular permeability through ectocervical cells is increased by two or more times due to combined TV and CST-IV bacteria activity [102].

### 4.1. Lactobacilli

*Lactobacilli* engage in a host-protective commensal relationship with their host, including through the production of lactic acid and antimicrobial compounds [103,104]. When *Lactobacilli* dominate the vaginal microbiome, protonated lactic acid protects the host against infection. Studying *Lactobacilli*-secreted lactic acid may prove useful in prophylactic and therapeutic contexts [104]. *Lactobacilli* are uniquely populous in the human vagina compared to other mammals [105,106]. Healthy vaginal microbiotas comprise primarily of these bacteria [106]. Various hypotheses attempt to explain this phenomenon, with one factor being the high amounts of glycogen available to sustain *Lactobacilli* [105]. Four of these hypotheses explain the bacterial levels as resulting from: (1) the continuous human ovarian cycle, (2) different bacteria filling their protective role in other mammals, (3) human promiscuity, and (4) a need to protect against infection via trauma to the uterus and vagina during childbirth [105]. *L. gasseri* aggregation-promoting factor APF-2 inhibits TV adhesion to ectocervical cells [107]. The bacteria secrete EVs containing proteins, nucleic acids (particularly RNA), lipids, and metabolites, modulating TV-host interactions [99]. *L. gasseri* can reduce TV-cell adhesion [108]. TV can manipulate *Lactobacillus* in its own favor, however, and is known to exploit similar bacteria to enhance disease progression [109]. TV has acquired nine fully functional NlpC/P60 family bacterial peptidoglycan-degrading enzymes through independent lateral gene transfer (LGT) events. These enzymes are upregulated in the presence of bacteria and degrade bacterial cell walls [110]. *T. vaginalis* expanded the NlpC/P60 genes into two distinct TVNlpC clans, with complementary functions. Exogenous expression of these enzymes allows TV to take over populations of *Lactobacilli* [111].

### 4.2. Gardnerella Vaginalis, Mycoplasma, and Prevotella Bivia in Bacterial Vaginosis

The biofilm produced by dysbiotic bacteria can enhance TV adhesion to host cells [112]. The adherent biofilm produced in BV is formed mainly by *Gardnerella vaginalis*. *G. vaginalis* is the predominant bacterium involved in BV, though it may not be the cause of the disease [13]. Some *G. vaginalis* strains do not form biofilms, depending partially on the expression of the sialidase A gene [113]. This biofilm attracts subsequent microbial colonizers, including TV [112]. Like Lactobacilli, *G. vaginalis* utilizes exosomes to regulate host–pathogen interactions [99]. *G. vaginalis* induces IL-8 and RANTES responses from the host, which can also be increased with activity from TV and *Atopobium vaginae*, leading to an inflammatory pathway facilitating additional infections, such as herpes simplex virus, human papillomavirus, and HIV [101].

TV participates in symbiosis with *Mycoplasma hominis* in the first and only known case of two endosymbiotic obligate human parasites able to produce infections in the same anatomical site and cause different diseases [114]. Any causal relationship between the two is unknown. *M. hominis* is another bacterium responsible for BV in the CST-IV microbiome [12]. *M. hominis* upregulates the proinflammatory response of monocytes to TV in vitro, affects mucosal integrity of the epithelium, has possible pro-cancerous effects, and has an influence on TV metabolism, particularly of arginine [114,115]. TV also protects *M. hominis* from antibiotics and host immune responses and may help transport the bacteria to its host [12,116]. TV infection is limited to the vagina, but by transporting and protecting *M. hominis*, which can infect the amniotic fluid, may represent a risk factor for pregnancy complications [12]. Candidatus *Mycoplasma girerdii* is a newly discovered TV-dependent symbiont [114]. Ca. *M. girerdii* is an unculturable bacterium that requires a CST-IV microbiome with an abundance of Prevotella [117]. *Prevotella bivia* is another BV pathobiont, that works with TV to dysregulate miRNA EV cargo corresponding to cancer, infectious disease, circadian rhythm, steroid hormone signaling, pregnancy, and reproductive tissue terms [118].

### 4.3. Trichomonasvirus (TVV)

TVV distribution in TV can affect the strengths of other diseases [119]. The presence of TVV has been demonstrated to enhance the inflammatory reaction to TV over 30-fold in vitro [101]. Five species of TVV can be found in the parasite, in various combinations that significantly influence its pathology [120]. TV immunity is suppressed more by TVV-positive parasites compared to TVV-negative phenotypes. TVV-positive exosomes exhibit limited IL-8, IL-6, and TNF-α cytokine secretion from mononuclear leukocytes, and decreased activity of the NF-κB, IL-8, and RANTES pathways in uterine endocervical cells [4]. TVV supports and influences the replicatory ability of TV, as well as other organisms in the microbiome [8]. In its normal lifecycle, TVV is only known to reproduce through vertical transmission, having no extracellular phase [71]. However, the viruses contained in TV can be released upon lysis of the parasite, allowing for additional infections to take root [50,120]. TVEVs may also transmit TVV to host cells, though the mechanism of transport is not clear. The presence of TVV in the vesicles alters their cargo, allowing TVV to draw a stronger immune response from the invaded cell [119].

The vaginal epithelial cells may release immunosuppressive small EVs in response to TVV-infected parasites [4]. These immunosuppressive effects can be replaced by immune stimulation once the parasite no longer hosts TVV [4]. TVV is also suspected to bind to TLR3, or to RIG-I and MDA5, and *M. hominis* binds to TLR2 [121,122].

### 4.4. HPV

TV is more prevalent in HPV-infected women than non-infected women. There are many hypotheses as to the association of the two infections. TV may catalyze HPV infection through various cytotoxic enzymes and the degradation of the basement membrane. Alternatively, TV infection may determine the increased risk of HPV persistence by inducing an inflammatory response in cervical-vaginal epithelial cells, damaging vaginal epithelial cells, cervical mucus, and IgA. There are few studies on the mechanism of TV influencing HPV infection [123]. TVAP65 hijacks signal peptidase complex subunit 1 (SPCS1) to transcriptionally upregulate CD151 and HSPG2. This could potentially be used as a therapeutic target and mitigate cervical cancer [124].

### 4.5. HIV

HIV contraction rates have been repeatedly shown to increase with TV infection, and as one meta-analysis found by 1.5 times [2,14,50]. Attacks by TV on the vaginal epithelium could facilitate the passage of HIV-1 to the underlying tissue [125]. Studies generally indicate that TV and HIV coinfection increases HIV shedding, allowing for spread to increase [14,126]. The IL-8 and RANTES pathways mentioned before may lead to carcinogenesis [101]. The immune response to TV in the presence of infectious HIV-1 may lead to increased HIV replication [125]. Galectin released from epithelial cells in response to TV may exacerbate diseases such as HIV [57]. TLR3 senses TVV, inducing an innate response and pro-inflammatory cascade also linked to preterm birth and greater susceptibility to HIV-1 [127].

### 4.6. Cancers

Cervical and prostate cancer are associated with TV infection [128,129]. Studies have reported varying risks of prostate cancer associated with TV infection [130,131]. The secretion of a TV homolog to human macrophage migration factor (MIF), a proinflammatory cytokine able to promote oncogenesis of prostate cancer, may be a mechanism for prostate tumor growth [114,129]. Adhesin contact with prostate cells may trigger a cascade altering known proto-oncogenes, PIM1, c-MYC, and HMGA1, leading to carcinogenesis [15]. Carcinogenesis is also promoted by inflammation caused by trichomoniasis and subsequent angiogenesis [15,130]. Preterm birth is caused by inflammation, breaking of the cervical barrier, and movement of pathogens into the upper reproductive tract, resulting from cervical cancer and many of the above diseases responsible for the same conditions [132].

## 5. Part 4. Discussion

### 5.1. Potential Therapeutic Targets of Nitroimidazole-Resistant TV Strains

The drug metronidazole and other 5-nitroimidazoles are often prescribed as remedies for trichomoniasis [2,55]. Unfortunately, multiple studies have found resistant TV strains that survive metronidazole treatment, and patients allergic to the medicine [71,133]. Ineffective metronidazole treatment may be supplemented with tinidazole or another drug. TV CPs may represent a possible target for chemotherapy and vaccine candidates instead of metronidazole [64]. Rhomboid serine proteases may be studied to investigate their similar potential [134]. Recombinant α-actinin subunit antigens of TV may pose as potential vaccine candidates, as ACT-F and ACT-T proteins were found to trigger strong immune responses and protect against TV infection [73]. A new technique for combating TV infection using nano-liposomal metronidazole may prove a useful substitute, given the rise in resistance to the drug, and provided further research is conducted on the new treatment [135]. Recent research has explored the inhibition of proteosomes as a method of killing TV, and there is ongoing work into identifying the best subunits to target [136].

The antibiotic metronidazole releases TVV virions when degrading TV. TVV virions and infected parasites enhance dsRNA-dependent pro-inflammatory responses, which may increase inflammation in the epithelium when metronidazole is used; however, the exact role of TVV in drug resistance remains poorly understood [137]. In the worst cases, resistance to this drug can allow some of the foulest outcomes of *Trichomonas* infection, such as pre-term delivery, to reach fruition. With concern for the increasing number of diseases becoming immune to current medicines, documented resistance should be addressed.

### 5.2. Questions, and Future Research on TV

The current number of quantitative analyses of the internalization of pathogen-derived EVs is limited [31]. Understudied alternative functions for some adhesin proteins may exist, as have been found with AP65 [39]. The signaling capabilities for the cytokines released in TV infection are not fully explored either and may prelude another immune or inflammatory response of some value. Leukotriene-B4, which is used by neutrophils to promote the attack of TV, is expressed in TV itself as well, which is stated to promote neutrophil activity, but also to prevent an inflammatory response via NOX2-mediated exocytotic degranulation in human mast cells [1,138]. The chemokine IP-10/CXCL10 is correlated with increased susceptibility to HIV-1 and so may be worth investigating [91]. TV contains a high number of BspA genes, though the mechanism of their adhesion performance is unknown [139]. Future studies are necessary on host receptors and pathways involved in symptomatic and asymptomatic sequelae, specifically the mechanisms behind BV and TV interactions, about which little is known [57,111].

Cancer poses a persistent threat to human health in nearly all fields of biology. The connection between cancer and TV is primarily through inflammation. Cases of cervical and prostate cancers have shown inconsistent associations with TV, as studies vary in their conclusions regarding a potential link. Further investigation is needed to clarify any underlying mechanisms and to better understand the nature of these correlations [130,140]. Continued investigation into the side effects of TV-stimulated inflammation may reveal connections to additional diseases. Research into the pseudocystic stage of TV has grown recently, despite being regarded as simply a degenerate stage not long ago. This stage may represent an area of interest for future studies on the assumed life cycle of the parasite and of other trichomonads. A recent study found that, though less so than trophozoites, pseudocysts seem to adhere to epithelial cells [141]. Recently, a viable cyst-like structure of TV was identified. This structure was physiologically robust, possibly enabling non-sexual transmission of TV. The parasite survived detergent, as well as chlorinated pool water in this form. When contacting host cells again, TV excysts back into a trophozoite [142]. A significant difference in the proteome between trophozoites and cysts exists. The parasite is also suggested to enter this phase when exposed to acidic vaginal pH [142].

TV infection is often asymptomatic, allowing cases to go unreported, meaning the already high estimations for TV cases may underrepresent the prevalence of the disease. TV spreads easily, and has grown to a global scale, with high concentrations in Africa and the Americas [2]. There exists a disparity in the affected demographics. Black communities are disproportionately affected by trichomoniasis, receiving reduced access to healthcare and inferior standards of care [143]. Over half of multiple Black inner-city populations are infected in the USA, with prevalence in reproductive age humans delivering serious reproductive consequences [144]. In addition to preterm birth, trichomoniasis is associated with low birth weight and premature membrane ruptures [144]. The effects of trichomoniasis demand serious attention, and with the advent of new hurdles to fighting the disease, the stagnation in TV research must be disrupted.

## 6. Conclusions

*Trichomonas vaginalis* remains a globally prevalent non-viral sexually transmitted pathogen whose pathogenesis relies on a multifaceted network of cytoadherence mechanisms, host immune modulation, and complex interactions with the vaginal microbiome and co-infections. Recent advances in understanding adhesins, lipophosphoglycan, cysteine proteinases, and extracellular vesicles have shed light on how TV establishes colonization, promotes immune evasion, and enhances inflammatory responses. The recognition of endosymbiotic partners such as *Mycoplasma hominis* and *Trichomonasvirus* further underscores the importance of considering TV not as an isolated pathogen but as part of a dynamic microbial consortium that exacerbates reproductive morbidity and susceptibility to HIV and cancers.

Despite these insights, critical gaps persist in elucidating the precise molecular pathways governing host–parasite interactions, the variability of symptomatic versus asymptomatic infections, and the mechanisms underlying drug resistance. Nitroimidazole resistance continues to undermine standard treatment, highlighting the urgent need for alternative therapeutic approaches, including protease inhibitors, recombinant vaccines, and nanotechnology-based drug delivery systems. Moreover, the potential oncogenic and reproductive health implications of TV infections demand broader epidemiological surveillance and mechanistic research.

In conclusion, integrating molecular parasitology, immunology, and microbiome science provides a more comprehensive picture of TV pathobiology. Continued investment in multidisciplinary research will be essential not only to clarify unanswered biological questions but also to translate these findings into innovative strategies for diagnosis, prevention, and therapy against this neglected yet highly consequential pathogen.

## Data Availability

No new data were created or analyzed in this study. All data used to support the findings of this study are available from the corresponding authors, Chang, J.-F. and Yeh, T.-Y., upon reasonable request.

## References

[1] Bhakta S.B., Moran J.A., Mercer F. (2020). Neutrophil interactions with the sexually transmitted parasite *Trichomonas vaginalis*: Implications for immunity and pathogenesis. Open Biol..

[2] World Health Organization Trichomoniasis. https://www.who.int/news-room/fact-sheets/detail/trichomoniasis.

[3] Petrin D., Delgaty K., Bhatt R., Garber G. (1998). Clinical and microbiological aspects of *Trichomonas vaginalis*. Clin. Microbiol. Rev..

[4] Govender Y., Chan T., Yamamoto H.S., Budnik B., Fichorova R.N. (2020). The Role of Small Extracellular Vesicles in Viral-Protozoan Symbiosis: Lessons From *Trichomonasvirus* in an Isogenic Host Parasite Model. Front. Cell Infect. Microbiol..

[5] Edwards T., Burke P., Smalley H., Hobbs G. (2016). *Trichomonas vaginalis*: Clinical relevance, pathogenicity and diagnosis. Crit. Rev. Microbiol..

[6] Paintlia M.K., Kaur S., Gupta I., Ganguly N.K., Mahajan R.C., Malla N. (2002). Specific IgA response, T-cell subtype and cytokine profile in experimental intravaginal trichomoniasis. Parasitol. Res..

[7] Mercer F., Johnson P.J. (2018). *Trichomonas vaginalis*: Pathogenesis, Symbiont Interactions, and Host Cell Immune Responses. Trends Parasitol..

[8] Zubacova Z., Cimburek Z., Tachezy J. (2008). Comparative analysis of trichomonad genome sizes and karyotypes. Mol. Biochem. Parasitol..

[9] Afzan M.Y., Suresh K. (2012). Pseudocyst forms of from cervical neoplasia. Parasitol. Res..

[10] Molgora B.M., Mukherjee S.K., Baumel-Alterzon S., Santiago F.M., Muratore K.A., Sisk A.E., Mercer F., Johnson P.J. (2023). *Trichomonas vaginalis* adherence phenotypes and extracellular vesicles impact parasite survival in a novel in vivo model of pathogenesis. PLoS Negl. Trop. Dis..

[11] Pereira-Neves A., Benchimol M. (2007). Phagocytosis by *Trichomonas vaginalis*: New insights. Biol. Cell.

[12] Margarita V., Fiori P.L., Rappelli P. (2020). Impact of Symbiosis Between *Trichomonas vaginalis* and *Mycoplasma hominis* on Vaginal Dysbiosis: A Mini Review. Front. Cell Infect. Microbiol..

[13] Patterson J.L., Stull-Lane A., Girerd P.H., Jefferson K.K. (2010). Analysis of adherence, biofilm formation and cytotoxicity suggests a greater virulence potential of *Gardnerella vaginalis* relative to other bacterial-vaginosis-associated anaerobes. Microbiology.

[14] Masha S.C., Cools P., Sanders E.J., Vaneechoutte M., Crucitti T. (2019). *Trichomonas vaginalis* and HIV infection acquisition: A systematic review and meta-analysis. Sex. Transm. Infect..

[15] Sutcliffe S., Neace C., Magnuson N.S., Reeves R., Alderete J.F. (2012). Trichomonosis, a common curable STI, and prostate carcinogenesis—A proposed molecular mechanism. PLoS Pathog..

[16] Leitsch D. (2021). Recent advances in the molecular biology of the protist parasite *Trichomonas vaginalis*. Fac. Rev..

[17] Hsu H.M., Yang Y.Y., Huang Y.H., Chu C.H., Tu T.J., Wu Y.T., Chiang C.J., Yang S.B., Hsu D.K., Liu F.T. (2023). Distinct features of the host-parasite interactions between nonadherent and adherent *Trichomonas vaginalis* isolates. PLoS Negl. Trop. Dis..

[18] Arroyo R., Gonzalez-Robles A., Martinez-Palomo A., Alderete J.F. (1993). Signalling of *Trichomonas vaginalis* for amoeboid transformation and adhesion synthesis follows cytoadherence. Mol. Microbiol..

[19] Noël C.J., Diaz N., Sicheritz-Ponten T., Safarikova L., Tachezy J., Tang P., Fiori P.L., Hirt R.P. (2010). vast BspA-like gene family: Evidence for functional diversity from structural organisation and transcriptomics. BMC Genom..

[20] Ortiz S., Verdan R., Fortes F., Benchimol M. (2023). *Trichomonas vaginalis*: Monolayer and Cluster Formation-Ultrastructural Aspects Using High-Resolution Scanning Electron Microscopy. Pathogens.

[21] Lizarraga A., Munoz D., Strobl-Mazzulla P.H., de Miguel N. (2021). Toward incorporating epigenetics into regulation of gene expression in the parasite *Trichomonas vaginalis*. Mol. Microbiol..

[22] Pachano T., Nievas Y.R., Lizarraga A., Johnson P.J., Strobl-Mazzulla P.H., de Miguel N. (2017). Epigenetics regulates transcription and pathogenesis in the parasite *Trichomonas vaginalis*. Cell Microbiol..

[23] Yeh Y.M., Huang K.Y., Richie Gan R.C., Huang H.D., Wang T.C., Tang P. (2013). Phosphoproteome profiling of the sexually transmitted pathogen *Trichomonas vaginalis*. J. Microbiol. Immunol. Infect..

[24] Nievas Y.R., Vashisht A.A., Corvi M.M., Metz S., Johnson P.J., Wohlschlegel J.A., de Miguel N. (2018). Protein Palmitoylation Plays an Important Role in *Trichomonas vaginalis* Adherence. Mol. Cell Proteom..

[25] Twu O., de Miguel N., Lustig G., Stevens G.C., Vashisht A.A., Wohlschlegel J.A., Johnson P.J. (2013). *Trichomonas vaginalis* exosomes deliver cargo to host cells and mediate host: Parasite interactions. PLoS Pathog..

[26] Nievas Y.R., Coceres V.M., Midlej V., de Souza W., Benchimol M., Pereira-Neves A., Vashisht A.A., Wohlschlegel J.A., Johnson P.J., de Miguel N. (2018). Membrane-shed vesicles from the parasite *Trichomonas vaginalis*: Characterization and their association with cell interaction. Cell Mol. Life Sci..

[27] Jahnke K., Staufer O. (2024). Membranes on the move: The functional role of the extracellular vesicle membrane for contact-dependent cellular signalling. J. Extracell. Vesicles.

[28] Kochanowsky J.A., Mira P.M., Elikaee S., Muratore K., Rai A.K., Riestra A.M., Johnson P.J. (2024). *Trichomonas vaginalis* extracellular vesicles up-regulate and directly transfer adherence factors promoting host cell colonization. Proc. Natl. Acad. Sci. USA.

[29] Ong S.C., Luo H.W., Cheng W.H., Ku F.M., Tsai C.Y., Huang P.J., Lee C.C., Yeh Y.M., Lin R., Chiu C.H. (2024). The core exosome proteome of *Trichomonas vaginalis*. J. Microbiol. Immunol. Infect..

[30] Salas N., Coceres V.M., Melo T.D.S., Pereira-Neves A., Maguire V.G., Rodriguez T.M., Sabatke B., Ramirez M.I., Sha J., Wohlschlegel J.A. (2021). VPS32, a member of the ESCRT complex, modulates adherence to host cells in the parasite *Trichomonas vaginalis* by affecting biogenesis and cargo sorting of released extracellular vesicles. Cell Mol. Life Sci..

[31] Rai A.K., Johnson P.J. (2019). *Trichomonas vaginalis* extracellular vesicles are internalized by host cells using proteoglycans and caveolin-dependent endocytosis. Proc. Natl. Acad. Sci. USA.

[32] Molgora B.M., Rai A.K., Sweredoski M.J., Moradian A., Hess S., Johnson P.J. (2021). A Novel *Trichomonas vaginalis* Surface Protein Modulates Parasite Attachment via Protein:Host Cell Proteoglycan Interaction. mBio.

[33] Salas N., Blasco Pedreros M., Dos Santos Melo T., Maguire V.G., Sha J., Wohlschlegel J.A., Pereira-Neves A., de Miguel N. (2023). Role of cytoneme structures and extracellular vesicles in *Trichomonas vaginalis* parasite-parasite communication. Elife.

[34] Rabinovich G.A., Gruppi A. (2005). Galectins as immunoregulators during infectious processes: From microbial invasion to the resolution of the disease. Parasite Immunol..

[35] Addis M.F., Rappelli P., Fiori P.L. (2000). Host and tissue specificity of *Trichomonas vaginalis* is not mediated by its known adhesion proteins. Infect. Immun..

[36] Dos Santos O., Rigo G.V., Macedo A.J., Tasca T. (2017). *Trichomonas vaginalis* clinical isolates: Cytoadherence and adherence to polystyrene, intrauterine device, and vaginal ring. Parasitol. Res..

[37] Hirt R.P., Noel C.J., Sicheritz-Ponten T., Tachezy J., Fiori P.L. (2007). *Trichomonas vaginalis* surface proteins: A view from the genome. Trends Parasitol..

[38] Crouch M.L., Alderete J.F. (1999). *Trichomonas vaginalis* interactions with fibronectin and laminin. Microbiology.

[39] Garcia A.F., Alderete J. (2007). Characterization of the *Trichomonas vaginalis* surface-associated AP65 and binding domain interacting with trichomonads and host cells. BMC Microbiol..

[40] Arroyo R., Engbring J., Alderete J.F. (1992). Molecular basis of host epithelial cell recognition by *Trichomonas vaginalis*. Mol. Microbiol..

[41] Rada P., Kellerova P., Verner Z., Tachezy J. (2019). Investigation of the Secretory Pathway in *Trichomonas vaginalis* Argues against a Moonlighting Function of Hydrogenosomal Enzymes. J. Eukaryot. Microbiol..

[42] Meza-Cervantez P., Gonzalez-Robles A., Cardenas-Guerra R.E., Ortega-Lopez J., Saavedra E., Pineda E., Arroyo R. (2011). Pyruvate:ferredoxin oxidoreductase (PFO) is a surface-associated cell-binding protein in *Trichomonas vaginalis* and is involved in trichomonal adherence to host cells. Microbiology.

[43] Chen Y.P., Riestra A.M., Rai A.K., Johnson P.J. (2019). A Novel Cadherin-like Protein Mediates Adherence to and Killing of Host Cells by the Parasite *Trichomonas vaginalis*. mBio.

[44] Lehker M.W., Arroyo R., Alderete J.F. (1991). The regulation by iron of the synthesis of adhesins and cytoadherence levels in the protozoan *Trichomonas vaginalis*. J. Exp. Med..

[45] Ardalan S., Lee B.C., Garber G.E. (2009). *Trichomonas vaginalis*: The adhesins AP51 and AP65 bind heme and hemoglobin. Exp. Parasitol..

[46] Zhang Z., Deng Y., Sheng W., Song X., Li Y., Li F., Pan Y., Tian X., Yang Z., Wang S. (2023). The interaction between adhesion protein 33 (TVAP33) and BNIP3 mediates the adhesion and pathogenicity of *Trichomonas vaginalis* to host cells. Parasit. Vectors.

[47] Zhang Z., Li Y., Wang S., Hao L., Zhu Y., Li H., Song X., Duan Y., Sang Y., Wu P. (2020). The Molecular Characterization and Immunity Identification of *Trichomonas vaginalis* Adhesion Protein 33 (AP33). Front. Microbiol..

[48] Fichorova R.N., Yamamoto H.S., Fashemi T., Foley E., Ryan S., Beatty N., Dawood H., Hayes G.R., St-Pierre G., Sato S. (2016). *Trichomonas vaginalis* Lipophosphoglycan Exploits Binding to Galectin-1 and -3 to Modulate Epithelial Immunity. J. Biol. Chem..

[49] Ryan C.M., de Miguel N., Johnson P.J. (2011). *Trichomonas vaginalis*: Current understanding of host-parasite interactions. Essays Biochem..

[50] Aquino M.F., Simoes-Barbosa A. (2022). A Microbial Pinata: Bacterial Endosymbionts of *Trichomonas vaginalis* Come in Different Flavors. mBio.

[51] Singh B.N., Hayes G.R., Lucas J.J., Sommer U., Viseux N., Mirgorodskaya E., Trifonova R.T., Sassi R.R., Costello C.E., Fichorova R.N. (2009). Structural details and composition of *Trichomonas vaginalis* lipophosphoglycan in relevance to the epithelial immune function. Glycoconj. J..

[52] Fichorova R.N., Trifonova R.T., Gilbert R.O., Costello C.E., Hayes G.R., Lucas J.J., Singh B.N. (2006). *Trichomonas vaginalis* lipophosphoglycan triggers a selective upregulation of cytokines by human female reproductive tract epithelial cells. Infect. Immun..

[53] Singh B.N. (1993). Lipophosphoglycan-like glycoconjugate of *Tritrichomonas foetus* and *Trichomonas vaginalis*. Mol. Biochem. Parasitol..

[54] Singh B., Lucas J., Fichorova R., Khan N.A. (2007). Emerging Protozoan Pathogens.

[55] Dylan D. (2017). Identification of Key Host Proteins Governing *Trichomonas vaginalis*: A Host-Parasite Interaction Study. Master’s Thesis.

[56] Okumura C.Y., Baum L.G., Johnson P.J. (2008). Galectin-1 on cervical epithelial cells is a receptor for the sexually transmitted human parasite *Trichomonas vaginalis*. Cell Microbiol..

[57] Fichorova R.N. (2009). Impact of *T. vaginalis* infection on innate immune responses and reproductive outcome. J. Reprod. Immunol..

[58] Arroyo R., Alderete J.F. (1989). *Trichomonas vaginalis* surface proteinase activity is necessary for parasite adherence to epithelial cells. Infect. Immun..

[59] Rendón-Gandarilla F.J., Ramón-Luing L.D., Ortega-López J., de Andrade I.R., Benchimol M., Arroyo R. (2013). The TvLEGU-1, a Legumain-Like Cysteine Proteinase, Plays a Key Role in Cytoadherence. Biomed. Res. Int..

[60] Zimmann N., Rada P., Zarsky V., Smutna T., Zahonova K., Dacks J., Harant K., Hrdy I., Tachezy J. (2022). Proteomic Analysis of *Trichomonas vaginalis* Phagolysosome, Lysosomal Targeting, and Unconventional Secretion of Cysteine Peptidases. Mol. Cell Proteom..

[61] Li Y., Li F., Tian W., Zhang Y., Wang W., Yang Z., Tian X., Wang S., Mei X., Zhang Z. (2024). Establishment of a programmatic detection method for Trichomonas vaginalis based on double antibody sandwich ELISA targeting TVCP39 antigen. Acta Trop..

[62] Mei X., Sheng W., Wang H., Tian W., Zhang Y., Zhou J., Zhang H., Song Z., Liu P., Han Y. (2025). TVCP39 is a key molecule in the pathogenicity and the synergy with human papillomavirus infection of Trichomonas vaginalis. Int. J. Biol. Macromol..

[63] Aranda-Chan V., Gutierrez-Soto M., Flores-Pucheta C.I., Montes-Flores O., Arroyo R., Ortega-Lopez J. (2025). Trichocystatin-2 from *Trichomonas vaginalis*: Role of N-terminal cysteines in aggregation, protease inhibition, and trichomonal cysteine protease-dependent cytotoxicity on HeLa cells. Front. Parasitol..

[64] Hernandez H.M., Marcet R., Sarracent J. (2014). Biological roles of cysteine proteinases in the pathogenesis of *Trichomonas vaginalis*. Parasite.

[65] Alderete J.F., Benchimol M., Lehker M.W., Crouch M.L. (2002). The complex fibronectin--*Trichomonas vaginalis* interactions and Trichomonosis. Parasitol. Int..

[66] Chang J.H., Ryang Y.S., Morio T., Lee S.K., Chang E.J. (2004). *Trichomonas vaginalis* inhibits proinflammatory cytokine production in macrophages by suppressing NF-kappaB activation. Mol. Cells.

[67] Alvarez-Sanchez M.E., Avila-Gonzalez L., Becerril-Garcia C., Fattel-Facenda L.V., Ortega-Lopez J., Arroyo R. (2000). A novel cysteine proteinase (CP65) of *Trichomonas vaginalis* involved in cytotoxicity. Microb. Pathog..

[68] Min D.Y., Hyun K.H., Ryu J.S., Ahn M.H., Cho M.H. (1998). Degradations of human immunoglobulins and hemoglobin by a 60 kDa cysteine proteinase of *Trichomonas vaginalis*. Korean J. Parasitol..

[69] Arroyo R., Cardenas-Guerra R.E., Figueroa-Angulo E.E., Puente-Rivera J., Zamudio-Prieto O., Ortega-Lopez J. (2015). *Trichomonas vaginalis* Cysteine Proteinases: Iron Response in Gene Expression and Proteolytic Activity. Biomed. Res. Int..

[70] Alderete J.F., Provenzano D. (1997). The vagina has reducing environment sufficient for activation of *Trichomonas vaginalis* cysteine proteinases. Genitourin. Med..

[71] Luo H.W., Ong S.C., Syu J.W., Tsai C.Y., Huang P.J., Lee C.C., Yeh Y.M., Lin R., Chiu C.H., Tang P. (2025). Dynamic and Heterogeneous Distribution of *Trichomonasvirus* Species in *Trichomonas vaginalis*. bioRxiv.

[72] Nemati M., Malla N., Yadav M., Khorramdelazad H., Jafarzadeh A. (2018). Humoral and T cell-mediated immune response against trichomoniasis. Parasite Immunol..

[73] Xie Y.T., Gao J.M., Wu Y.P., Tang P., Hide G., Lai D.H., Lun Z.R. (2017). Recombinant alpha-actinin subunit antigens of *Trichomonas vaginalis* as potential vaccine candidates in protecting against trichomoniasis. Parasit. Vectors.

[74] Miranda-Ozuna J.F., Hernandez-Garcia M.S., Brieba L.G., Benitez-Cardoza C.G., Ortega-Lopez J., Gonzalez-Robles A., Arroyo R. (2016). The Glycolytic Enzyme Triosephosphate Isomerase of *Trichomonas vaginalis* Is a Surface-Associated Protein Induced by Glucose That Functions as a Laminin- and Fibronectin-Binding Protein. Infect. Immun..

[75] Lama A., Kucknoor A., Mundodi V., Alderete J.F. (2009). Glyceraldehyde-3-phosphate dehydrogenase is a surface-associated, fibronectin-binding protein of *Trichomonas vaginalis*. Infect. Immun..

[76] Munoz C., Perez M., Orrego P.R., Osorio L., Gutierrez B., Sagua H., Castillo J.L., Martinez-Oyanedel J., Arroyo R., Meza-Cervantez P. (2012). A protein phosphatase 1 gamma (PP1gamma) of the human protozoan parasite *Trichomonas vaginalis* is involved in proliferation and cell attachment to the host cell. Int. J. Parasitol..

[77] Ryu J.S., Choi H.K., Min D.Y., Ha S.E., Ahn M.H. (2001). Effect of iron on the virulence of *Trichomonas vaginalis*. J. Parasitol..

[78] Wu K.Y., Chen Y.J., Lin S.F., Hsu H.M. (2025). Iron triggers PI4P5K proteostasis and Arf-mediated cell membrane trafficking to regulate PIP signaling crucial for multiple pathogenic activities of the parasitic protozoan. Mbio.

[79] Chen Y.J., Wu K.Y., Lin S.F., Huang S.H., Hsu H.C., Hsu H.M. (2023). PIP2 regulating calcium signal modulates actin cytoskeleton-dependent cytoadherence and cytolytic capacity in the protozoan parasite *Trichomonas vaginalis*. PLoS Pathog..

[80] Alderete J.F. (1999). Iron modulates phenotypic variation and phosphorylation of P270 in double-stranded RNA virus-infected *Trichomonas vaginalis*. Infect. Immun..

[81] Handrich M.R., Garg S.G., Sommerville E.W., Hirt R.P., Gould S.B. (2019). Characterization of the BspA and Pmp protein family of trichomonads. Parasit. Vectors.

[82] Riestra A.M., Gandhi S., Sweredoski M.J., Moradian A., Hess S., Urban S., Johnson P.J. (2015). A Rhomboid Protease and Its Substrate Modulate Parasite Attachment and Cytolysis of Host Cells. PLoS Pathog..

[83] Malla N., Yadav M., Gupta I. (2007). Kinetics of serum and local cytokine profile in experimental intravaginal trichomoniasis induced with *Trichomonas vaginalis* isolates from symptomatic and asymptomatic women. Parasite Immunol..

[84] Riestra A.M., Valderrama J.A., Patras K.A., Booth S.D., Quek X.Y., Tsai C.M., Nizet V. (2019). *Trichomonas vaginalis* Induces NLRP3 Inflammasome Activation and Pyroptotic Cell Death in Human Macrophages. J. Innate Immun..

[85] Uribe-Querol E., Rosales C. (2021). The Multiple Roles of Trogocytosis in Immunity, the Nervous System, and Development. BioMed Res. Int..

[86] Mercer F., Ng S.H., Brown T.M., Boatman G., Johnson P.J. (2018). Neutrophils kill the parasite *Trichomonas vaginalis* using trogocytosis. PLoS Biol..

[87] Ryu J.S., Kang J.H., Jung S.Y., Shin M.H., Kim J.M., Park H., Min D.Y. (2004). Production of interleukin-8 by human neutrophils stimulated with *Trichomonas vaginalis*. Infect. Immun..

[88] Frasson A.P., Menezes C.B., Goelzer G.K., Gnoatto S.C.B., Garcia S.C., Tasca T. (2017). Adenosine reduces reactive oxygen species and interleukin-8 production by *Trichomonas vaginalis*-stimulated neutrophils. Purinergic Signal.

[89] Han I.H., Goo S.Y., Park S.J., Hwang S.J., Kim Y.S., Yang M.S., Ahn M.H., Ryu J.S. (2009). Proinflammatory cytokine and nitric oxide production by human macrophages stimulated with *Trichomonas vaginalis*. Korean J. Parasitol..

[90] Yang J.B., Quan J.H., Kim Y.E., Rhee Y.E., Kang B.H., Choi I.W., Cha G.H., Yuk J.M., Lee Y.H. (2015). Involvement of PI3K/AKT and MAPK Pathways for TNF-alpha Production in SiHa Cervical Mucosal Epithelial Cells Infected with *Trichomonas vaginalis*. Korean J. Parasitol..

[91] Liu C.C., Chu L.J., Yeh Y.M., Lin H.C., Chen L.C., Huang C.Y., Chiu S.F., Cheng F.W., Lin W.N., Huang K.Y. (2025). Immunomodulatory roles of autophagic flux and IFIT in human ectocervical cells upon *Trichomonas vaginalis* infection. Int. Immunopharmacol..

[92] Huang C.Y., Chiu S.F., Chu L.J., Yeh Y.M., Huang P.J., Lan K.L., Tsai Y.L., Liu C.C., Chen C.F., Lin W.N. (2025). *Trichomonas vaginalis* extracellular vesicles activate the NLRP3 inflammasome and TLR3-mediated inflammatory cascades in host cells. PLoS Pathog..

[93] Lin M.C., Hui C.F., Chen J.Y., Wu J.L. (2012). The antimicrobial peptide, shrimp anti-lipopolysaccharide factor (SALF), inhibits proinflammatory cytokine expressions through the MAPK and NF-kappaB pathways in *Trichomonas vaginalis* adherent to HeLa cells. Peptides.

[94] Gao F.F., Quan J.H., Lee M.A., Ye W., Yuk J.M., Cha G.H., Choi I.W., Lee Y.H. (2021). *Trichomonas vaginalis* induces apoptosis via ROS and ER stress response through ER-mitochondria crosstalk in SiHa cells. Parasit. Vectors.

[95] Menezes S.A., Cardoso F.G., Venturi C.R., Barden A.T., Tasca T. (2025). Adenosine deprivation modulates purine metabolism and enhances *Trichomonas vaginalis* cytotoxicity. Biochimie.

[96] Natto M.J., Miyamoto Y., Munday J.C., AlSiari T.A., Al-Salabi M.I., Quashie N.B., Eze A.A., Eckmann L., De Koning H.P. (2021). Comprehensive characterization of purine and pyrimidine transport activities in *Trichomonas vaginalis* and functional cloning of a trichomonad nucleoside transporter. Mol. Microbiol..

[97] Mercer F., Diala F.G., Chen Y.P., Molgora B.M., Ng S.H., Johnson P.J. (2016). Leukocyte Lysis and Cytokine Induction by the Human Sexually Transmitted Parasite *Trichomonas vaginalis*. PLoS Negl. Trop. Dis..

[98] Kaur S., Khurana S., Bagga R., Wanchu A., Malla N. (2008). Antitrichomonas IgG, IgM, IgA, and IgG subclass responses in human intravaginal trichomoniasis. Parasitol. Res..

[99] Artuyants A., Hong J., Dauros-Singorenko P., Phillips A., Simoes-Barbosa A. (2024). *Lactobacillus gasseri* and *Gardnerella vaginalis* produce extracellular vesicles that contribute to the function of the vaginal microbiome and modulate host-*Trichomonas vaginalis* interactions. Mol. Microbiol..

[100] Moodley P., Connolly C., Sturm A.W. (2002). Interrelationships among human immunodeficiency virus type 1 infection, bacterial vaginosis, trichomoniasis, and the presence of yeasts. J. Infect. Dis..

[101] Fichorova R.N., Buck O.R., Yamamoto H.S., Fashemi T., Dawood H.Y., Fashemi B., Hayes G.R., Beach D.H., Takagi Y., Delaney M.L. (2013). The villain team-up or how *Trichomonas vaginalis* and bacterial vaginosis alter innate immunity in concert. Sex. Transm. Infect..

[102] Hinderfeld A.S., Phukan N., Bar A.K., Roberton A.M., Simoes-Barbosa A. (2019). Cooperative Interactions between *Trichomonas vaginalis* and Associated Bacteria Enhance Paracellular Permeability of the Cervicovaginal Epithelium by Dysregulating Tight Junctions. Infect. Immun..

[103] Younes J.A., Lievens E., Hummelen R., van der Westen R., Reid G., Petrova M.I. (2018). Women and Their Microbes: The Unexpected Friendship. Trends Microbiol..

[104] O’Hanlon D.E., Moench T.R., Cone R.A. (2013). Vaginal pH and microbicidal lactic acid when lactobacilli dominate the microbiota. PLoS ONE.

[105] Miller E.A., Beasley D.E., Dunn R.R., Archie E.A. (2016). Lactobacilli Dominance and Vaginal pH: Why Is the Human Vaginal Microbiome Unique?. Front. Microbiol..

[106] Zhou J.Z., Way S.S., Chen K. (2018). Immunology of Uterine and Vaginal Mucosae: (Trends in Immunology 39, 302–314, 2018). Trends Immunol..

[107] Phukan N., Brooks A.E.S., Simoes-Barbosa A. (2018). A Cell Surface Aggregation-Promoting Factor from *Lactobacillus gasseri* Contributes to Inhibition of *Trichomonas vaginalis* Adhesion to Human Vaginal Ectocervical Cells. Infect. Immun..

[108] Phukan N., Parsamand T., Brooks A.E., Nguyen T.N., Simoes-Barbosa A. (2013). The adherence of *Trichomonas vaginalis* to host ectocervical cells is influenced by *lactobacilli*. Sex. Transm. Infect..

[109] Cardoso F.G., Tasca T. (2025). Advancements in vaginal microbiota, *Trichomonas vaginalis*, and vaginal cell interactions: Insights from co-culture assays. Microb. Cell.

[110] Pinheiro J., Biboy J., Vollmer W., Hirt R.P., Keown J.R., Artuyants A., Black M.M., Goldstone D.C., Simoes-Barbosa A. (2018). The Protozoan Trichomonas vaginalis Targets Bacteria with Laterally Acquired NlpC/P60 Peptidoglycan Hydrolases. mBio.

[111] Barnett M.J., Pinheiro J., Keown J.R., Biboy J., Gray J., Lucinescu I.W., Vollmer W., Hirt R.P., Simoes-Barbosa A., Goldstone D.C. (2023). NlpC/P60 peptidoglycan hydrolases of Trichomonas vaginalis have complementary activities that empower the protozoan to control host-protective lactobacilli. PLoS Pathog..

[112] Hinderfeld A.S., Simoes-Barbosa A. (2020). Vaginal dysbiotic bacteria act as pathobionts of the protozoal pathogen *Trichomonas vaginalis*. Microb. Pathog..

[113] Hardy L., Jespers V., Van den Bulck M., Buyze J., Mwambarangwe L., Musengamana V., Vaneechoutte M., Crucitti T. (2017). The presence of the putative *Gardnerella vaginalis* sialidase A gene in vaginal specimens is associated with bacterial vaginosis biofilm. PLoS ONE.

[114] Dessi D., Margarita V., Cocco A.R., Marongiu A., Fiori P.L., Rappelli P. (2019). *Trichomonas vaginalis* and *Mycoplasma hominis*: New tales of two old friends. Parasitology.

[115] Fiori P.L., Diaz N., Cocco A.R., Rappelli P., Dessi D. (2013). Association of *Trichomonas vaginalis* with its symbiont *Mycoplasma hominis* synergistically upregulates the in vitro proinflammatory response of human monocytes. Sex. Transm. Infect..

[116] Dessi D., Delogu G., Emonte E., Catania M.R., Fiori P.L., Rappelli P. (2005). Long-term survival and intracellular replication of *Mycoplasma hominis* in *Trichomonas vaginalis* cells: Potential role of the protozoon in transmitting bacterial infection. Infect. Immun..

[117] Martin D.H., Zozaya M., Lillis R.A., Myers L., Nsuami M.J., Ferris M.J. (2013). Unique vaginal microbiota that includes an unknown *Mycoplasma*-like organism is associated with *Trichomonas vaginalis* infection. J. Infect. Dis..

[118] Cezar-de-Mello P.F.T., Ryan S., Fichorova R.N. (2023). The microRNA Cargo of Human Vaginal Extracellular Vesicles Differentiates Parasitic and Pathobiont Infections from Colonization by Homeostatic Bacteria. Microorganisms.

[119] Rada P., Hrdy I., Zdrha A., Narayanasamy R.K., Smutna T., Horackova J., Harant K., Benes V., Ong S.C., Tsai C.Y. (2022). Double-Stranded RNA Viruses Are Released From Trichomonas vaginalis Inside Small Extracellular Vesicles and Modulate the Exosomal Cargo. Front. Microbiol..

[120] Hetzel C.A., Appah-Sampong A.A., Hurst-Manny A.R., Nibert M.L. (2024). Differential Drug Susceptibility across *Trichomonasvirus* Species Allows for Generation of Varied Isogenic Clones of *Trichomonas vaginalis*. Pathogens.

[121] Jensen S., Thomsen A.R. (2012). Sensing of RNA viruses: A review of innate immune receptors involved in recognizing RNA virus invasion. J. Virol..

[122] Love W., Dobbs N., Tabor L., Simecka J.W. (2010). Toll-like receptor 2 (TLR2) plays a major role in innate resistance in the lung against murine *Mycoplasma*. PLoS ONE.

[123] Mei X., Zhang R., Li D., Xie X., Yao Y., Gao M., Zhao L., Zhu S., Tian X., Yang Z. (2023). Association between the infections of Trichomonas vaginalis and uterine cervical human papillomavirus: A meta-analysis. J. Obstet. Gynaecol..

[124] Mei X., Sheng W., Zhang Y., Tian W., Tian X., Yang Z., Wang S., Zhang Z. (2025). *Trichomonas vaginalis* adhesion protein 65 facilitates human papillomavirus entry via SPCS1-mediated upregulation of CD151 and HSPG2 in keratinocyte lineage. Infect. Dis. Poverty.

[125] Guenthner P.C., Secor W.E., Dezzutti C.S. (2005). *Trichomonas vaginalis*-induced epithelial monolayer disruption and human immunodeficiency virus type 1 (HIV-1) replication:: Implications for the sexual transmission of HIV-1. Infect. Immun..

[126] Kissinger P., Amedee A., Clark R.A., Dumestre J., Theall K.P., Myers L., Hagensee M.E., Farley T.A., Martin D.H. (2009). *Trichomonas vaginalis* treatment reduces vaginal HIV-1 shedding. Sex. Transm. Dis..

[127] Hirt R.P. (2013). virulence factors: An integrative overview. Sex. Transm. Infect..

[128] Yap E.H., Ho T.H., Chan Y.C., Thong T.W., Ng G.C., Ho L.C., Singh M. (1995). Serum antibodies to *Trichomonas vaginalis* in invasive cervical cancer patients. Genitourin. Med..

[129] Twu O., Dessi D., Vu A., Mercer F., Stevens G.C., de Miguel N., Rappelli P., Cocco A.R., Clubb R.T., Fiori P.L. (2014). *Trichomonas vaginalis* homolog of macrophage migration inhibitory factor induces prostate cell growth, invasiveness, and inflammatory responses. Proc. Natl. Acad. Sci. USA.

[130] Shui I.M., Kolb S., Hanson C., Sutcliffe S., Rider J.R., Stanford J.L. (2016). *Trichomonas vaginalis* infection and risk of advanced prostate cancer. Prostate.

[131] Kim S.S., Kim J.H., Han I.H., Ahn M.H., Ryu J.S. (2016). Inflammatory Responses in a Benign Prostatic Hyperplasia Epithelial Cell Line (BPH-1) Infected with *Trichomonas vaginalis*. Korean J. Parasitol..

[132] Daskalakis G., Psarris A., Koutras A., Fasoulakis Z., Prokopakis I., Varthaliti A., Karasmani C., Ntounis T., Domali E., Theodora M. (2023). Maternal Infection and Preterm Birth: From Molecular Basis to Clinical Implications. Children.

[133] Klebanoff M.A., Carey J.C., Hauth J.C., Hillier S.L., Nugent R.P., Thom E.A., Ernest J.M., Heine R.P., Wapner R.J., Trout W. (2001). Failure of metronidazole to prevent preterm delivery among pregnant women with asymptomatic *Trichomonas vaginalis* infection. N. Engl. J. Med..

[134] Riestra A.M. (2015). Characterization of *Trichomonas vaginalis* Rhomboid Proteases and Surface Proteins that Contribute to Host: Parasite Interactions. Ph.D. Thesis.

[135] Ebrahimi M., Montazeri M., Ahmadi A., Nami S., Hamishehkar H., Shahrivar F., Bakhtiar N.M., Nissapatorn V., Spotin A., Ahmadpour E. (2021). Nanoliposomes increases Anti-*Trichomonas vaginalis* and apoptotic activities of metronidazole. Acta Trop..

[136] Fajtova P., Hurysz B.M., Miyamoto Y., Serafim M., Jiang Z., Trujillo D.F., Liu L., Somani U., Almaliti J., Myers S.A. (2023). Development of subunit selective substrates for *Trichomonas vaginalis* proteasome. bioRxiv.

[137] Fichorova R.N., Lee Y., Yamamoto H.S., Takagi Y., Hayes G.R., Goodman R.P., Chepa-Lotrea X., Buck O.R., Murray R., Kula T. (2012). Endobiont viruses sensed by the human host—Beyond conventional antiparasitic therapy. PLoS ONE.

[138] Min A., Lee Y.A., Kim K.A., El-Benna J., Shin M.H. (2017). SNAP23-Dependent Surface Translocation of Leukotriene B4 (LTB4) Receptor 1 Is Essential for NOX2-Mediated Exocytotic Degranulation in Human Mast Cells Induced by *Trichomonas vaginalis*-Secreted LTB4. Infect. Immun..

[139] Zhao Y., Lian Y., Guan W., Wu P., Yang S., Li J. (2024). Chromosome-level genome assembly, reannotation and decoding of a *Trichomonas vaginalis* clinical isolate from Shiyan, Central China. Decod. Infect. Transm..

[140] World Health Organization (2024). New Treatment Recommendations. Recommendations for the Treatment of Trichomonas vaginalis, Mycoplasma genitalium, Candida albicans, Bacterial vaginosis and Human papillomavirus (Anogenital Warts).

[141] Dias-Lopes G., Saboia-Vahia L., Margotti E.T., Fernandes N.S., Castro C.L.F., Oliveira F.O.J., Peixoto J.F., Britto C., Silva F.C.E.F., Cuervo P. (2017). Morphologic study of the effect of iron on pseudocyst formation in *Trichomonas vaginalis* and its interaction with human epithelial cells. Mem. Inst. Oswaldo Cruz.

[142] Beri D., Yadav P., Devi H.R.N., Narayana C., Gadara D., Tatu U. (2019). Demonstration and Characterization of Cyst-Like Structures in the Life Cycle of *Trichomonas vaginalis*. Front. Cell Infect. Microbiol..

[143] Bassey G.B., Clarke A.I.L., Elhelu O.K., Lee C.M. (2022). Trichomoniasis, a new look at a common but neglected STI in African descendance population in the United States and the Black Diaspora. A review of its incidence, research prioritization, and the resulting health disparities. J. Natl. Med. Assoc..

[144] Shafir S.C., Sorvillo F.J., Smith L. (2009). Current issues and considerations regarding trichomoniasis and human immunodeficiency virus in African-Americans. Clin. Microbiol. Rev..

